# 

**Published:** 1888-09

**Authors:** 


					﻿SEPTEMBER, 1888.
OLD SERIES
Vouxibe^
NEW SERIES
Vol. V-No. 7.
& l85S-'v'*


ejOIMNAIi
l^x et	>


W.F.WESTMORELAND.M Dj
H.V.M.MILLER.M.D.LL.D.
Published ky james p.harrison&c®
Weu	Atlanta,ga, M
SUBSCRIPTION t S2.DO A YEAR.
				

